# Proposal of a screening instrument for autism spectrum disorder in children (Mini-TEA Scale)

**DOI:** 10.1055/s-0044-1780517

**Published:** 2024-03-04

**Authors:** Cassiano Mateus Forcelini, Regina Ampese, Helena Younes de Melo, Camila Pereira Neubauer Pasin, José Renato Donadussi Pádua, Camila Boschetti Spanholo, Francine Ehrhardt Hoffmann, Júlia Breitenbach Diniz, Laís Cristine Zanella Capponi, Luiza Souza, Maxciel Zortea

**Affiliations:** 1 Associação de Pais e Amigos dos Excepcionais (APAE), Passo Fundo RS, Brazil.; 2 Universidade de Passo Fundo, Escola de Medicina, Passo Fundo RS, Brazil.; 3 Universidade do Valo do Rio dos Sinos, São Leopoldo RS, Brazil.

**Keywords:** Autism Spectrum Disorder, Triage, Sensitivity and Specificity, Transtorno do Espectro Autista, Triagem, Sensibilidade e Especificidade

## Abstract

**Background** Autism spectrum disorder (ASD) requires trained professionals for its adequate diagnosis. There is a shortage of such professionals in Brazil. Screening tools could identify priority cases. The only instrument for that in Brazilian Portuguese is employed for toddlers up to 2.5 years old.

**Objective** The Mini-TEA scale was conceived and tested as a screening for children from 2.5 to 12 years old.

**Methods** After local ethics committee's approval, this study was conducted from December 2022 to April 2023 in the *Associação de Pais e Amigos dos Excepcionais* , Passo Fundo/RS, of invitations to children's parents/relatives who were under evaluation for ASD and by local advertisement. Inclusion criteria were age from 2.5 to 12 years old; consent from the child's legal guardians. 75 children's parents/relatives were interviewed using the 15-item Mini-TEA scale. After that, children were evaluated for the diagnosis of ASD by a pediatric neurologist. Sensibility and specificity for ASD diagnosis along the Mini-TEA scores were measured. Experts and target population evaluated the validity/reliability of the Mini-TEA scale. The reproducibility of the scores was assessed about 40 days later.

**Results** From the 75 participants, 28 received a diagnosis of ASD. Scores ≥ 10 on the Mini-TEA scale require further evaluation of the children (sensitivity 100%; specificity 68%). Content validity coefficient (CVC) rendered values > 0.80 (acceptable). Test-retest analyzes with the intraclass correlation coefficient (ICC) indicated excellent reliability (> 0.90). The time spent for applying the screening was about 10 minutes.

**Conclusion** The Mini-TEA scale presents as an easy tool for screening ASD among children.

## Resumo

**Antecedentes** O transtorno do espectro autista (TEA) requer profissionais treinados para o diagnóstico, escassos no Brasil. Instrumentos de triagem poderiam identificar casos prioritários para avaliação. O único em português brasileiro é empregado para crianças até 30 meses de idade.

**Objetivo** A escala Mini-TEA foi concebida e testada como triagem para crianças entre 2,5 e 12 anos.

**Métodos** Estudo foi conduzido de dezembro de 2022 a abril de 2023 na Associação de Pais e Amigos dos Excepcionais (APAE) de Passo Fundo/RS, após a aprovação bioética local. O recrutamento consistiu em convite aos familiares de crianças que estavam sendo avaliadas para TEA e por divulgação local. Os critérios de inclusão foram idade entre 2,5 e 12 anos e consentimento do guardião legal. Familiares de 75 crianças foram entrevistados com a escala Mini-TEA (15 itens). Depois, as crianças foram avaliadas para o diagnóstico de TEA por neuropediatra. A sensibilidade e a especificidade do diagnóstico de TEA com os escores da Mini-TEA foram mensuradas. A validade e a confiabilidade da escala Mini-TEA foram avaliadas por experts e pela população alvo. A reprodutibilidade dos escores foi medida após ± 40 dias.

**Resultados** Dos 75 participantes, 28 receberam diagnóstico de TEA. Escores ≥ 10 na escala Mini-TEA requerem avaliação das crianças (sensibilidade 100%; especificidade 68%). O coeficiente de validação de conteúdo (CVC) rendeu valores > 0,80 (aceitável). Análises de teste-reteste com coeficiente de correlação intraclasse (ICC) indicou excelente confiabilidade (> 0,90). O tempo gasto para a triagem foi cerca de 10 minutos.

**Conclusão** A escala Mini-TEA constitui ferramenta breve e fácil para triagem de TEA em crianças.

## INTRODUCTION

Autism spectrum disorder (ASD) comprises a conjunct of neurodevelopmental disabilities usually first evident in infancy or childhood. The diagnostic criteria require persistent deficits in social communication and social interaction across multiple contexts, as well as restricted, repetitive patterns of behavior, interests, or activities, all these symptoms causing impairment in social, occupational, or other important areas of current functioning. 1

The most robust data about the frequency of ASD periodically come from the USA, among children aged eight years. The survey undertaken in 2020 pointed to one in 36 children (approximately 4% of boys and 1% of girls). 2 These estimates are higher than previous studies performed during 2000-2018, 2 suggesting that the frequency and/or the diagnosis of ASD is growing. This highlights the need for enhanced infrastructure to provide diagnostic, treatment, and support services for all children with ASD, 2 a condition with unknown prevalence in Brazil.

Several diagnostic instruments for ASD are available, some of them translated and validated into Brazilian Portuguese or even originally formulated in Brazil. 3 4 However, there is a scarcity of trained professionals for the adequate diagnosis of neurodevelopmental disabilities in Brazil, similar to other developing countries. 5 This gives rise to the suitability of screening tools with enough diagnostic accuracy to separate those infants and children who actually need further evaluation from those whose suspicion of ASD is not appropriate, rationalizing the use of limited health resources. On the other hand, a screening instrument applied to a large extent would be a step towards early diagnosis, a golden opportunity for offering a variety of evidence-based interventions that confer a better prognosis. 6 7

The widespread use of the Modified Checklist for Autism (M-CHAT) in toddlers has been a recommendation for pediatricians because there is empirical support for its utility in population screening. 8 The revised form of this checklist (M-CHAT-R/F) was recently translated and validated into Brazilian Portuguese. 9 However, this instrument is directed to toddlers from 16 to 30 months of age, a population that needs easy access to pediatric care in developing countries. As a result, most preschool-aged children and school students were not assessed for ASD, and there is a lack of a simple tool for evaluating them in Brazil. In this setting, we designed a screening scale (Mini-TEA) for ASD ( *transtorno do espectro autista* ) in Brazilian Portuguese, but directed to parents/relatives of children from 2.5 to 12 years old. The diagnostic accuracy and reproducibility of this instrument were assessed and are presented in this study, along with other characteristics related to the validation process and feasibility.

## METHODS

### Research site

This study was conducted from December 2022 to April 2023 in the *Associação de Pais e Amigos dos Excepcionais* (APAE), Passo Fundo, RS, Brazil, an institution devoted to assistance to disabled people. Since April 2022, the APAE from Passo Fundo houses a *Centro Regional de Referência em Transtorno do Espectro Autista* (Regional Reference Center for ASD) of the *Programa TEAcolhe,* a program for improving diagnosis and management of ASD supported by the Government of Rio Grande do Sul (RS), Brazil. The local ethics committee approved the protocol in December 2022 (approval number 5.800.005).

### Population

The recruitment comprised direct invitations to children and their parents/relatives who were under evaluation for possible ASD, as well as to those attracted by local advertisement. The inclusion criteria were:

- child aged from 2.5 to 12 years old;
- consent from the child's legal guardians and, whenever feasible, from the child.

The exclusion criterion was guardians' illiteracy. No subject declined participation.

### Study protocol, measures, and outcomes

Upon the written consent, the participants underwent the following protocol:

- Obtainment of demographic and clinical data from an interview with each subject and his parents/relatives;
- Application of the Mini-TEA scale to the parents/relatives by medical students;
- Clinical evaluation of the child by a pediatric neurologist, accompanied by the parents/relatives, regarding the diagnostic criteria of ASD from the DSM-V-RV, 1 and application of the Childhood Autism Rating Scale (CARS). 3 The pediatric neurologist remained unaware of the patients' scores on the Mini-TEA scale until the end of the study. In parallel, the medical students who applied the scale also remained blinded regarding the patients' final diagnoses and CARS scores.

Ultimately, the results were assessed to define the primary outcome: the cut-off point on the Mini-TEA score that could offer a higher sensitivity for screening to ASD, considering the diagnostic criteria according to DSM-V-RV as the gold standard. Predefined secondary outcomes were CARS scores, intra and inter-observer reproducibility, and interview duration for applying the Mini-TEA. The latter was recorded as an estimative of the time spent screening for ASD.

A sample composed of the first 30 parents/relatives was resubmitted to the Mini-TEA scale between one and two months later to assess the reproducibility. They were randomly interviewed by the same medical student (intra-observer correlation: 15 parents/relatives) or by a different medical student (inter-observer correlation: 15 parents/relatives).

### Structure of the Mini-TEA scale

We designed the Mini-TEA scale as a screening tool to be applied to children's parents/relatives during an interview. The scale was built inspired by two previous instruments for assessing ASD: the M-CHAT and the CARS. We combined the objectivity of the M-CHAT, with questions accepting only “yes” or “no”, with an assessment divided into 14 items that correspond to different groups of symptoms, as that performed by a trained professional during the execution of the CARS, which requires the presence of the child with the parents/relatives. In the 15 ^th^ item, the diagnostic impression from who applies the CARS was substituted with parents'/relatives' impression about the presence or absence of abnormal neurodevelopment. Moreover, each of the former 14 items consists of 2 to 5 binary questions, with any positive answer making the item score “1”, independently from the number of positive answers in the item. The item's score points to “0” only if all its questions contain a negative answer. Thus, the 15-item Mini-TEA scale ranges from 0 to 15 and was originally comprised of 51 questions. In case more than one parent/relative is contributing to the answers, and a discrepancy arises between them (“yes” vs. “no”), the higher score is considered (“yes”).

### Validity and reliability of the Mini-TEA scale

An analysis of validity and reliability was performed. Three experts (not involved in the conduction of the survey) were invited to score the quality of the items from 1 (not at all) to 5 (enough) regarding the following aspects:

- Comprehensibility: How comprehensible is this item for you, in terms of grammatical and syntactical language characteristics?
- Congruency: How much is the item close/accounting/a part of the ASD construct?
- Relevancy: How relevant is the item to the clinical practice of the diagnosis of ASD?

A target population of 10 children's parents/relatives was also assessed similarly about three characteristics:

- Comprehensibility: How comprehensible is this question for you?
- Congruency: How much does this question represent a description of the ASD patients?
- Difficulty/ease: How difficult was it to answer this question thinking about your child?

### Statistical analysis

The sample size was dimensioned for the factorial exploratory analysis as proposed by Kyriazos, 10 who concluded that at least 5 participants per item are necessary for factor estimating. This required at least 75 participants.

Mean, standard deviation, median, variation, standard error, frequency, and percentage were used for descriptive purposes of clinical and sociodemographic data and the primary measures of the study, according to the nature of the variable. Shapiro-Wilk normality tests and visual inspection of histograms were used to evaluate the distribution of the quantitative variables.

To examine the sources of validity evidence of the Mini-TEA scale, the following strategies were used: a) analysis by expert judges, b) pilot-administration study with a target population, c) convergence analysis with other variables, and d) test-criteria analysis with an external measure (gold standard). For a) and b) the content validity coefficient (CVC) was applied to each item, 11 which considers values above 0.80 acceptable. For c) Spearman's correlation coefficient ( *ρ* ) and explained variance obtained from univariate linear and quantile regression analyses between the CARS instrument (predictor variable) and Mini-TEA (criterion variable) scores were used. Analysis by quantiles, 12 a non-parametric strategy, allows the evaluation of the relationship between the measures at different levels of the criterion variable. For d), a ROC curve (receiver-operating curve) was drawn to verify levels of specificity and sensitivity of the Mini-TEA scores in identifying cases of ASD established with the DSM-V-TR. Likewise, this analysis allowed an estimate of the instrument cut-off point.

To test the reliability of the Mini-TEA scale, internal consistency analysis was investigated using the modified alpha coefficient (Kuder-Richardson coefficient, KR20), which considers dichotomous items (yes/no). Values above 0.90 indicate high internal consistency. Test-retest analyses were performed using the Intraclass Correlation Coefficient (ICC), applied separately for comparisons between inter-examiners and intra-examiners. For this purpose, F statistics and related p-values were used to test if ICC has a null or equal to zero value (H:0 → ICC = 0; H:1 → ICC ≠ 0). Values of ICC 0.75 or greater indicate acceptable agreement between applications.

## RESULTS

### Sociodemographic data

The sample comprised 75 children whose parents/relatives answered the Mini-TEA scale. All participants completed the study and were evaluated for the diagnosis of ASD and the score in the CARS. Table 1 presents detailed information about them.

**Table 1:** Sociodemographic and clinical data of the total sample (n = 75)

Continuous variables		N	Mean ± SD	Median (IQR)
Age (years)		75	6.79 ± 3.04	6.2 (4.2–9.4)
Administration time (minutes)		60	10.45 ± 2.67	10.0 (8.7–12.0)
Years of study		75	1.00 ± 1.73	0 (0–1.0)
Mini-TEA score (1st evaluation)		75	9.93 ± 5.23	12.0 (5.0–15.0)
Mini-TEA score (2 ^nd^ evaluation)		30	8.17 ± 6.36	9.0 (1.0–14.5)
CARS score		75	27.55 ± 11.96	23.0 (17.0–38.5)
			**Categories**	
**Categorical variables**		**N**	**Absolut count**	**%**
Sex	Male	75	56	74.67
	Female		19	25.33
Learning problems	Yes	75	45	60.00
	No		30	40.00
Behavior problems	Yes	75	55	73.33
	No		20	26.67
Speech problems	Yes	75	48	64.00
	No		27	36.00
ASD diagnosis	Yes	75	28	37.33
	No		47	62.67

Abbreviations: ASD, autism spectrum disorder; CARS, Childhood Autism Rating Scale; IQR, interquartile range; SD, standard deviation.

Of the 75 participants, 28 had the diagnosis of ASD confirmed. Learning, behavior, and speech problems were the leading symptoms that motivated the parents/relatives to seek aid. Yet, there were also volunteers without any complaints who contributed to the study sample after local advertising. Alternative diagnoses of ASD included intellectual disabilities, communication disorders, attention-deficit/hyperactivity disorder, and oppositional defiant disorder. These children were referred to medical accompaniment.

### Evidence of validity

#### *Analysis by experts*

Based on the analysis by the experts, the CVC values were obtained for each item. Table 2 depicts this data. All items were classified as acceptable, indicating that the experts recognized that the items are comprehensible, congruent to the construct, and relevant to the ASD diagnosis.

**Table 2:** Content validity coefficients (CVC) values based on the responses from 3 experts and 10 participants for judging the quality of the 15 items of Mini-TEA scale

	Analysis by experts (n = 3)			Evaluation by the target population (n = 10)		
Item	Comprehensibility	Congruency	Relevancy	Comprehensibility	Congruency	Difficulty
**1**	0.96	0.96	0.96	0.94	0.96	0.96
**2**	0.96	0.96	0.96	0.92	0.96	0.96
**3**	0.96	0.96	0.96	0.94	0.96	0.96
**4**	0.83	0.96	0.96	0.96	0.96	0.96
**5**	0.90	0.96	0.96	0.96	0.96	0.96
**6**	0.96	0.96	0.96	0.92	0.96	0.96
**7**	0.96	0.96	0.90	0.96	0.96	0.83
**8**	0.96	0.96	0.96	0.96	0.96	0.96
**9**	0.96	0.90	0.96	0.94	0.94	0.90
**10**	0.96	0.90	0.96	0.94	0.94	0.83
**11**	0.83	0.96	0.96	0.96	0.96	0.96
**12**	0.96	0.96	0.96	0.92	0.96	0.96
**13**	0.96	0.96	0.96	0.96	0.96	0.96
**14**	0.90	0.96	0.96	0.96	0.96	0.96
**15**	0.96	0.83	0.83	0.96	0.96	0.96

Notes: Values above 0.90 indicate high internal consistency, while values above from 0.80 to 0.90 are considered acceptable.

#### *Pilot application in a target population*

The analysis of the quality of the items made by a target population is shown in Table 2 based on the CVC calculated for each item. All exhibited high quality, given the criteria of comprehensibility, pertinence to the ASD construct, and difficulty/ease of answering were considered acceptable.

### Reliability analyses

#### *Internal consistency analysis*

A coefficient of KR20 = 0.95 (95% confidence interval = 0.93; 0.96) was observed for the 15 Mini-TEA scale items, indicating a very high internal consistency.

#### *Test-retest analysis*

The ICC for repeated tests indicated excellent instrument reliability with high coefficients. Table 3 depicts a summary of these results. The interval between applications varied from 27 to 61 days (mean = 40 days), without differences between groups (t test: inter and intra-examiners; p = 0.841).

**Table 3:** Test-retest analysis with intra-class correlation coefficient (ICC) between examinees and intra-examinees (total n = 30)

Group	N	CVC	F	*p*	95% CI
Inter-examiners	15	0.957	42.5	<0.001	0.877 < ICC < 0.985
Intra-examiners	15	0.931	35.6	<0.001	0.765 < ICC < 0.978

Abbreviation: CI, confidence interval. Notes: F statistics and related p-values were used to test if ICC has null or equal to zero value ((H:0 → ICC = 0; H:1 → ICC ≠ 0).

### Sensitivity and specificity analysis and cut-off point

**Figure 1:**
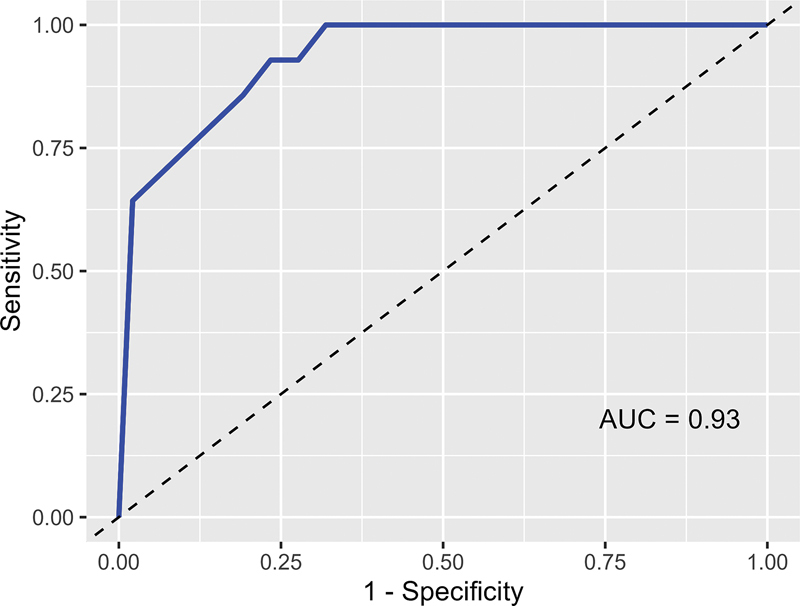
ROC curve for the sensitivity and specificity of Mini-TEA scale in predicting the diagnosis of autism spectrum disorder.

Table 4 presents the sensitivity and specificity of the Mini-TEA scale to predict cases and non-cases of ASD. As a screening test, when sensitivity was prioritized, the cut-off point to identify suspected ASD was proposed: scores equal to 10 or higher had 100% of sensitivity and 68% of specificity for the diagnosis. Figure 1 illustrates this through a ROC curve that presented an area under the curve (AUC) ROC value of 0.93, indicating the high discriminating quality.

**Table 4:** Sensitivity and specificity of the Mini-TEA scores to predict ASD cases

Score	Sensitivity	Specificity
15	0.00	1.00
14	0.64	0.98
13	0.86	0.81
12	0.93	0.77
11	0.93	0.72
10	1.00	0.68
9	1.00	0.64
8	1.00	0.55
7	1.00	0.51
6	1.00	0.47
5	1.00	0.38
4	1.00	0.34
3	1.00	0.21
2	1.00	0.15
1	1.00	0.02
0	1.00	0.00

Note: The cut-off point of ≥10 had 100% of sensitivity and 68% of specificity for the diagnosis of autism spectrum disorder (ASD).

### Convergent validity

**Figure 2:**
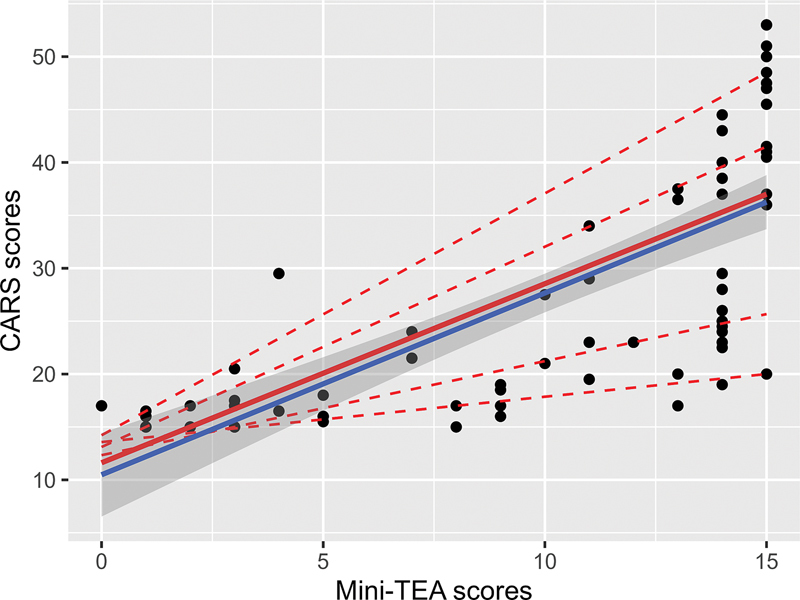
Convergent validity test through predictions of linear regression (blue) and quantile regression (red). In addition to the model for the 0.5 quantile or median (thicker line), four dashed lines represent the 0.1, 0.3, 0.7 and 0.9 quantiles. R ^2^ adjusted for the linear model: 0.56.

As the Mini-TEA scores were not normally distributed (W = 0.83, p = < 0.001), we decided to undertake both parametric and non-parametric regression analyses. Firstly, we found evidence for a strong positive association between Mini-TEA and CARS scores (ρ = 0.864, p < 0.005). For further investigation of this relation, quantile (for non-parametric data) and linear regression models showed that CARS scores are significantly related to the Mini-TEA scores, even across the different score quantiles. Table 5 pictures the regression coefficients for the generated models. CARS responses explained 55.76% of the Mini-TEA variance. Figure 2 demonstrates a scatterplot of the relation between CARS and Mini-TEA scores.

**Table 5:** Regression coefficients of quantile regression and linear regression models between Mini-TEA (criterion) and CARS (predictor) scores

Quantile	B	SE	T	p
Q: 0.1	0.37	0.03	12.53	<0.001
Q: 0.3	0.38	0.06	6.31	<0.001
Q: 0.5	0.38	0.03	13.88	<0.001
Q: 0.7	0.22	0.05	4.28	<0.001
Q: 0.9	0.06	>0.01	46.97	<0.001
Linear	0.33	0.03	9.71	<0.001

Abbreviation: SE, standard error.

### Final version of the instrument

We launched a quantitative analysis of the questions regarding the capacity to change the score of the items. In this sense, it was observed that three questions (in items 2, 3, and 12) did not discriminate the presence or absence of symptoms. Furthermore, some of these questions overlapped semantically with others of the same item. Thus, with the purpose of a brief screening, we decided to remove these questions from the instructions. The Supplementary Material ( https://www.arquivosdeneuropsiquiatria.org/wp-content/uploads/2023/12/ANP-2023.0150-Supplementary-Material.docx ) delivers the final version of the scale, with 48 questions distributed along the 15 items. The questionnaire of this final version probably takes about 10 minutes or less to be performed, because the original scale with 51 questions took a mean of 10.45 minutes.

## DISCUSSION

ASD is a condition that requires trained professionals for proper diagnosis. Similar to other developing countries, 5 Brazil lacks such professionals. Screening tools could help separate those children who need further evaluation from others, justifying the use of limited health resources. The only easy instrument for screening ASD in Brazilian Portuguese is the M-CHAT, which is progressively being operated by professionals devoted to the care of children as pediatricians. However, this instrument was tested in toddlers from 16 to 30 months of age, a population that often skips the opportunity to be assessed for ASD at that age period due to limited access to pediatric care in developing countries. 5 Although early identification of ASD has improved in Brazil, it still represents only about 30% of the diagnoses made. 13 This high proportion of late identification negatively impacts the patients' prognosis since it is established that early therapeutic intervention can improve the neurodevelopment in ASD. 6 7

In this scenario, we created the Mini-TEA scale to fill the gap. This study demonstrated preliminary results that suggest the helpfulness of applying the Mini-TEA scale in a variegated population of children aged 2.5 to 12 years old, providing information regarding its validation, reproducibility, and accuracy. The cut-off point of a score equal to 10 or higher in the Mini-TEA scale indicates the need for further evaluation because this had 100% sensitivity and reasonable specificity: no participant with ASD was missed, and 64% of those without ASD were ascertained of the non-diagnosis by a score up to 9. It should be noted that a cut-off point of 11 would lose seven percentage points of sensitivity and elevate specificity by only four percentage points. Therefore, we decided to maintain a lower score to ensure suspected cases would be referred to specialized services for an adequate diagnosis. Considering the evidence that the earlier the therapeutic interventions begin, the better the prognosis in ASD, 6 7 even if the diagnosis is still not confirmed, sensitivity should be prioritized.

The theme had motivated a similar study undertaken in India, 5 where a 37-item instrument in Hindi with dichotomous yes/no responses was developed to be applied to children aged 1.5-10 yr. Curiously, the results pointed to a score of 10 as a cut-off (sensitivity 89.16%; specificity 89.13%).

Screening methods have typically not been sufficiently sensitive in that they have not identified most children with ASD in general populations in whom parents have not already noticed a delay. 14 The Mini-TEA scale may be a strategy to face this issue because of the ease of application and interpretation, without the necessity of previous training or specific formation, short questionnaire duration, and the exemption of the child during the evaluation. This could turn screening purposes into feasible targets in primary medical attention and even in schools. Another potential utility could be selecting a sample of the population for prevalence studies, lacking information in Brazil.

A couple of limitations exist. The sample size is insufficient for defining critical questions or items that could express a higher probability of ASD diagnosis, like the current form of M-CHAT. This requires a larger survey with statistical power explicitly defined as a central outcome for this purpose. A larger sample could also bring more information about other intervening epidemiological factors that were not evaluated. This is a standard process in the improvement of a diagnostic tool. Indeed, M-CHAT has undergone a refinement process over the decades. 15 16 17 18

We recognize that the main limitation of this survey is its exploratory and preliminary nature, providing data from a limited series of children from a small geographical area located in Southern Brazil. Further studies with larger casuistries certainly could test the properties of the Mini-TEA scale, especially if considering populations from other country regions.

In summary, the Mini-TEA scale may be a valuable tool for screening ASD among children to increase early identification and treatment and consequently improve the prognosis of patients with ASD.
