# Supplementary material for: Proposal of a screening instrument for autism spectrum disorder in children (Mini-TEA Scale)
Source: Arq Neuropsiquiatr. 2024 Mar 4;82(3):s00441780517. doi: 10.1055/s-0044-1780517 (PMC10911887; doi:10.1055/s-0044-1780517)
Supplement: Supplementary file 1 — Supplementary Material [file 10-1055-s-0044-1780517-s230150.pdf]

Escala Mini-TEA para triagem de transtorno do espectro autista

**1. Relações pessoais:** [ ] 0 (nenhuma resposta SIM) [ ] 1 (pelo menos uma resposta SIM)
A criança evita olhar diretamente nos olhos?<br>Tem grande dificuldade em ter interação com adultos ou pessoas da mesma idade (timidez exagerada)?<br>Chega a ficar muito zangado ou desesperado com alguma tentativa de interação com adultos ou pessoas da mesma idade?<br>Mostra-se distante, ausente, como se não percebesse ou não importasse a outra pessoa?<br>É muito difícil conseguir a atenção da criança?
**2. Imitação:** [ ] 0 (nenhuma resposta SIM) [ ] 1 (pelo menos uma resposta SIM)
A criança tem dificuldade para imitar gestos simples, como bater palmas?<br>Tem dificuldade ou muita demora para imitar sons?<br>Só com muito estímulo a criança imita gestos ou sons?
**3. Resposta emocional:** [ ] 0 (nenhuma resposta SIM) [ ] 1 (pelo menos uma resposta SIM)
A criança às vezes tem reações emocionais aparentemente sem relação com o que o que está presente ou acontece à sua volta (ex: gritar ou rir sem motivo aparente)?<br>Ou costuma ser indiferente a situações em que se esperaria um reação?
**4. Movimentos corporais:** [ ] 0 (nenhuma resposta SIM) [ ] 1 (pelo menos uma resposta SIM)
A criança é muito desajeitada (descoordenada)?<br>Apresenta movimentos repetitivos?<br>Tem movimentos estranhos com os dedos das mãos ou com o corpo?<br>Costuma caminhar mais na ponta dos pés?<br>Ocorre de agredir a si mesma (bater-se, morder-se, etc)?
**5. Uso de objetos:** [ ] 0 (nenhuma resposta SIM) [ ] 1 (pelo menos uma resposta SIM)
A criança brinca de forma estranha com objetos, diferente do que seria esperado (ex: sugando, batendo, arrastando, enfileirando)?<br>Fixa a atenção somente em um aspecto ou parte do objeto ou brinquedo (ex: roda)?<br>Tem interesse restrito, ou seja, somente em um ou dois objetos?<br>Mostra pouco interesse em brinquedos e objetos?
**6. Adaptação à mudança:** [ ] 0 (nenhuma resposta SIM) [ ] 1 (pelo menos uma resposta SIM)
A criança tem muita dificuldade em aceitar mudar o que está fazendo para passar para outra atividade?<br>Fica zangada ou triste por mudanças simples (ex: lugar dos móveis, trajeto para escola)?<br>Quando ocorrem mudanças bruscas mostra uma reação intensa (é difícil de acalmar)?
**7. Resposta visual:** [ ] 0 (nenhuma resposta SIM) [ ] 1 (pelo menos uma resposta SIM)
A criança tem alguma forma estranha de olhar para alguns objetos (ex: de muito perto, só para uma parte do objeto)?<br>Apresenta momentos de olhar parado, para o nada, como se estivesse ausente?<br>Evita constantemente olhar para objetos apresentados?
**8. Resposta ao som:** [ ] 0 (nenhuma resposta SIM) [ ] 1 (pelo menos uma resposta SIM)
Parece que a criança às vezes não ouve direito ou não atende quando chamada pelo nome?<br>Tem reações inesperadas (ex: crise de irritabilidade, tapar os ouvidos) quando ouve alguns sons do ambiente?
**9. Resposta ao paladar, olfato e tato:**<br>[ ] 0 (nenhuma resposta SIM) [ ] 1 (pelo menos uma resposta SIM)
A criança segue levando à boca objetos, mesmo que outras crianças da sua idade já não façam mais isso?<br>Tenta cheirar ou experimentar o gosto de objetos não comestíveis, e até pessoas?<br>Gosta de comer só alguns alimentos específicos, recusando-se a experimentar outros?<br>A reação a um estímulo doloroso é fora do normal: quase não reage ou reage muito mais intensamente que o esperado?
**10. Medo ou nervosismo:** [ ] 0 (nenhuma resposta SIM) [ ] 1 (pelo menos uma resposta SIM)
Parece que a criança muitas vezes tem medo ou nervosismo exagerado?<br>Chega a ter medo de coisas inofensivas?<br>Ou parece não ter medo quando seria esperado que tivesse (ex: perto de cachorro latindo, atravessar a rua)?<br>Chega a ser difícil acalmar a criança nas crises de medo?
**11. Comunicação verbal:** [ ] 0 (nenhuma resposta SIM) [ ] 1 (pelo menos uma resposta SIM)
A criança está atrasada para falar?<br>Tem uma fala estranha, na escolha dos sons ou palavras, nem sempre fácil de entender?<br>Fica repetindo muito só coisas que vê na TV (ex: números em inglês)?

(*Continued*)

 © 2024. The Author(s).

(*Continued*)

**12. Comunicação não verbal:** [ ] 0 (nenhuma resposta SIM) [ ] 1 (pelo menos uma resposta SIM)
A criança tem dificuldade em se comunicar por gestos?<br>Tem dificuldade de entender os gestos ou as expressões faciais que fazemos?<br>Em vez de pegar algo ao seu alcance, puxa um adulto para pegar o objeto para ela?
**13. Nível de atividade:** [ ] 0 (nenhuma resposta SIM) [ ] 1 (pelo menos uma resposta SIM)
A criança tem inquietude maior que outras crianças da idade?<br>Chega a não conseguir parar quieta para fazer algo?<br>Ou é muito parada, lenta, preguiçosa perto de outras crianças da idade?
**14. Resposta intelectual:** [ ] 0 (nenhuma resposta SIM) [ ] 1 (pelo menos uma resposta SIM)
A criança parece ser menos esperta para aprender as coisas em relação às outras crianças?<br>Tem dificuldade de aprender especificamente só algumas coisas que outras da mesma idade já sabem?<br>Parece ser muito mais esperta em uma coisa específica do que as demais crianças da idade?
**15. Impressão dos familiares:** [ ] 0 (nenhuma resposta SIM) [ ] 1 (pelo menos uma resposta SIM)
Você acha que sua criança tem algo de diferente do usual ou atrasado em relação ao desenvolvimento, comparado com outras crianças?
**Escore final:**

Instruções:

A. Essa escala se destina ao(s) responsável(is) de crianças entre 2 anos e meio de idade e 12 anos completos.
B. O(s) entrevistado(s) deve(m) ser questionado(s) presencialmente por um entrevistador (não é auto-aplicável).
C. A escala tem 15 itens, cada um podendo pontuar "1" ou "0". No total, a pontuação mínima da escala é "0" e a máxima é "15".
D. Cada item tem de uma a cinco perguntas. Resposta "SIM" para quaisquer das perguntas, não importa quantas (uma, duas, todas, etc) leva à pontuação "1" no item. Se todas as respostas das perguntas do item forem "NÃO", a pontuação será "0".
E. Caso se aplique simultaneamente a escala em mais de uma pessoa (ex: ambos os pais) e houver discordância em questões ("sim" vs. "não"), levar em consideração a pontuação mais alta em cada pergunta ("sim").
F. Essa escala não é diagnóstica de Transtorno do Espectro Autista (TEA). Crianças com pontuação de 10 ou mais devem ser melhor avaliadas com relação à possibilidade de TEA.

 © 2024. The Author(s).
